# Multiple strategies of oxygen supply in *Drosophila* malignancies identify tracheogenesis as a novel cancer hallmark

**DOI:** 10.1038/srep09061

**Published:** 2015-03-12

**Authors:** Daniela Grifoni, Manuela Sollazzo, Elisabetta Fontana, Francesca Froldi, Annalisa Pession

**Affiliations:** 1 Department of “Farmacia e Biotecnologie”, University of Bologna, Bologna, Italy

## Abstract

Angiogenesis is the term used to describe all the alterations in blood vessel growth induced by a tumour mass following hypoxic stress. The occurrence of multiple strategies of vessel recruitment favours drug resistance, greatly complicating the treatment of certain tumours. In *Drosophila*, oxygen is conveyed to the internal organs by the tracheal system, a closed tubular network whose role in cancer growth is so far unexplored. We found that, as observed in human cancers, *Drosophila* malignant cells suffer from oxygen shortage, release pro-tracheogenic factors, co-opt nearby vessels and get incorporated into the tracheal walls. We also found that the parallelisms observed in cellular behaviours are supported by genetic and molecular conservation. Finally, we identified a molecular circuitry associated with the differentiation of cancer cells into tracheal cells. In summary, our findings identify tracheogenesis as a novel cancer hallmark in *Drosophila*, further expanding the power of the fly model in cancer research.

Cancer is a complex and extremely heterogeneous disease, and huge efforts have been made to devise a conceptual framework in which to gather the recurring phenotypic traits associated with tumour onset and progression^1^. The most part of these traits has been successfully investigated in cellular and animal models; in particular, thanks to the development of specific genetic tools, the fruitfly *Drosophila* *melanogaster* has greatly served the purpose. In the last decade, *Drosophila* models of epithelial cancers have indeed helped elucidate fundamental aspects of tumour biology, such as the mechanisms linking cell polarity to proliferation control^2^. The regulation of epithelial cell polarity is a crucial issue in cancer biology, as its loss correlates with invasive and metastatic abilities of cancer cells and is strongly associated with a poor prognosis^3^. In *Drosophila*, *lethal giant larvae* (*lgl*), *discs large* (*dlg*) and *scribble* (*scrib*) are a class of tumour suppressor genes (TSGs) that encode functionally conserved proteins involved in the maintenance of epithelial apical-basal polarity as well as in proliferation control^4^. Their loss-of-function (LOF) phenotypes are especially evident in the imaginal discs, larval epithelial organs that give rise to adult structures following metamorphosis, which show unrestrained growth, complete loss of tissue architecture and ability to fuse with nearby tissues, so displaying local invasiveness^4^. Despite the obvious malignant traits shown by the mutant organs, when mutation in one of these genes is induced clonally, that is in single cells within a wild-type tissue, such mutant cells undergo apoptotic death triggered by non cell-autonomous safeguard mechanisms named “cell competition”^5^ or “intrinsic tumour suppression”^6^. Since clonal induction is however the genetic system that best mimics mammalian cancer onset, several models of cooperative tumourigenesis have been established in *Drosophila* in which such mutations are combined with oncogenic Ras/Raf signalling. This pathway, whose activating mutations are found in a high percentage of human cancers, provides polarity-deficient cells with survival and proliferation properties^7^ necessary to overwhelm the surrounding wild-type tissue and form malignant masses^8^,^9^,^10^. In the imaginal cells, *Ras*^V12^ ectopic expression alone causes instead hyperplastic growth of the tissue^11^; malignant growth thus requires a functional cooperation between loss of cell polarity and hyperplasia. We previously demonstrated that *lgl* human orthologue, namely *Lgl1* or *Hugl-1*, functions as a tumour suppressor gene also in humans, and our and other studies described its altered expression/localisation in several forms of cancer^12^,^13^,^14^. Two pathways have been mainly implicated in *lgl*-driven malignant growth: the Hippo pathway and the JNK pathway. The Hippo (Hpo) pathway is a highly conserved signalling network that plays a key role in epithelial growth; it is composed of several upstream regulators which activity converge on the transcriptional co-activator Yorkie (Yki - YAP in humans)^15^,^16^. When the pathway is active, Yki is sequestered in the cytoplasm and, upon pathway deregulation, it translocates into the nucleus and activates the expression of several target genes involved in cell growth, proliferation and resistance to apoptosis both in *Drosophila*^17^ and humans^15^,^16^,^18^. In contexts in which the *lgl* mutation triggers tumour growth, the inactivation of the Hpo pathway is responsible for the major part of the malignant phenotype^2^. The JNK pathway is a conserved stress-induced MAPK cascade that has multiple roles in cellular processes including proliferation, differentiation, morphogenesis and apoptosis, and has been implicated in several aspects of cancer^19^. In *lgl* mutant cells grown in a wild-type background, which suffer from cell competition, the apoptotic death triggered by the neighbours has been demonstrated to be JNK-dependent^5^. Nevertheless, in *lgl*^−^; *Ras*^V12^ malignant clones the JNK pathway promotes growth and invasion, this latter mainly through the induction of matrix metalloproteinases (MMP), whose function is to break down the basement membrane underlining the epithelial sheet^20^,^21^. Cell polarity loss thus results in JNK activation which, in the presence of an active *Ras* signalling that restrains its pro-apoptotic effects, induces invasive behaviour and stimulates growth. The same effect was observed in imaginal discs in which *lgl* was downregulated by RNA interference; upon loss of cell polarity, cancer growth was shown to be JNK-dependent^22^. Among the cancer hallmarks described by Hanahan and Weinberg^1^, angiogenesis plays a fundamental role in tumour expansion. Growing masses suffer from oxygen shortage, and low oxygen tension leads to stabilisation and nuclear accumulation of the transcription factor HIF1α (Hypoxia-Inducible Factor 1α) through the inhibition of the prolyl-hydroxylases that prime it for degradation in normoxia^23^. HIF1α is a strong activator of the VEGF (Vascular Endothelial Growth Factor) promoter; secreted VEGF triggers sprouting angiogenesis (SA) by binding its receptor and activating the downstream signalling in endothelial cells^23^. Oncogenic *Ras* signalling is also able to upregulate VEGF, as well as other endothelial growth factors such as FGF^7^. In *Drosophila*, oxygen is conveyed to the internal organs through an interconnected tubular network called “tracheal system”, whose regulation is significantly analogue to that of mammalian angiogenesis, with *Drosophila* FGF/FGFR (FGF Receptor), encoded by the *branchless* (*bnl*) and *breathless* (*btl*) genes respectively, carrying out the functions of VEGF/VEGFR^24^,^25^. Further to drive the formation of the tracheal tree during embryonic and post-embryonic development, the FGF/FGFR signalling is also active during late larval development, when a structure connected to the basal side of the wing imaginal disc, the “air sac”, emerges from a tracheal branch named “transverse connective” and undergoes a morphogenetic process which relies on both proliferation and migration^26^. *Drosophila* tracheal network is also known to respond to local hypoxic conditions through a mechanism strikingly similar to that of mammalian SA: hypoxia is sensed by prolyl-hydroxylases, that are hence inactivated, and Similar (Sima), the *Drosophila* HIF1α, is thereby stabilised and in turn contributes to the induction of FGF expression to attract new terminal branches towards the hypoxic cells^25^,^27^. At all these stages, tracheal remodelling and elongation requires the expression of MMP1^28^. In addition to SA, alternative forms of vascular modification have been described in mammals, i.e. intussusceptive angiogenesis (IA), vascular co-option (VC) and vascular mimicry (VM)^29^. IA was first described in the 90s and consists in the splitting of pre-existing vessels with the resulting formation of microcapillaries; it is a process faster than SA, predominantly found in large tumours^30^. VC is a mechanism observed both in primary lesions and in metastases, by which tumours obtain oxygen supply hijacking the pre-existing vasculature; tumour cells migrate along the vessels of the organ and, as the mass increases, the vessels become completely embedded in the tumour^31^. VM is a process mainly detected in aggressive tumours in which cancer cells help form mosaic vessels characterised by alternated tumour and endothelial cell clusters, or compose a capillary network on their own; cancer stem-like cells can also trans-differentiate into endothelial cells^32^. Anti-angiogenic therapies have been developed based on the evidence that SA occurs upon VEGF secretion by hypoxic tumour cells; specific drugs have thus been designed to block VEGF signalling, but the occurrence of all these mechanisms alternative to SA greatly complicates the treatment of certain tumours^33^. There is indeed evidence that both primary tumours and metastases are able to progress without SA; such tumours have been described in lung, liver and lymph nodes^31^. By inducing oncogenesis in *Drosophila* epithelia we were able to observe several, distinguishable cellular strategies of oxygen supply whose morphological features overlap with those above described in mammalian cancers. We demonstrate that tumour masses are hypoxic, show Sima/HIF1α nuclear accumulation and FGF ectopic expression. Malignant cells are both able to co-opt pre-existing tracheal branches and to differentiate into tracheal cells and participate in the composition of mosaic vessels, recapitulating the behaviours associated with mammalian VC and VM. In addition, our data suggest that the epithelial-to-tracheal switch requires the regulation of Polycomb and STAT92E by the JNK signalling cascade. In summary, our findings identify tracheogenesis as a novel cancer hallmark in *Drosophila* and support the use of this model for the investigation of genetic and molecular alterations resulting from hypoxic stress in human cancers.

## Results

### Characterisation of the *Minute-ENgrailed* (*MEN)-lgl*^KD^ system

**Figure 1:**
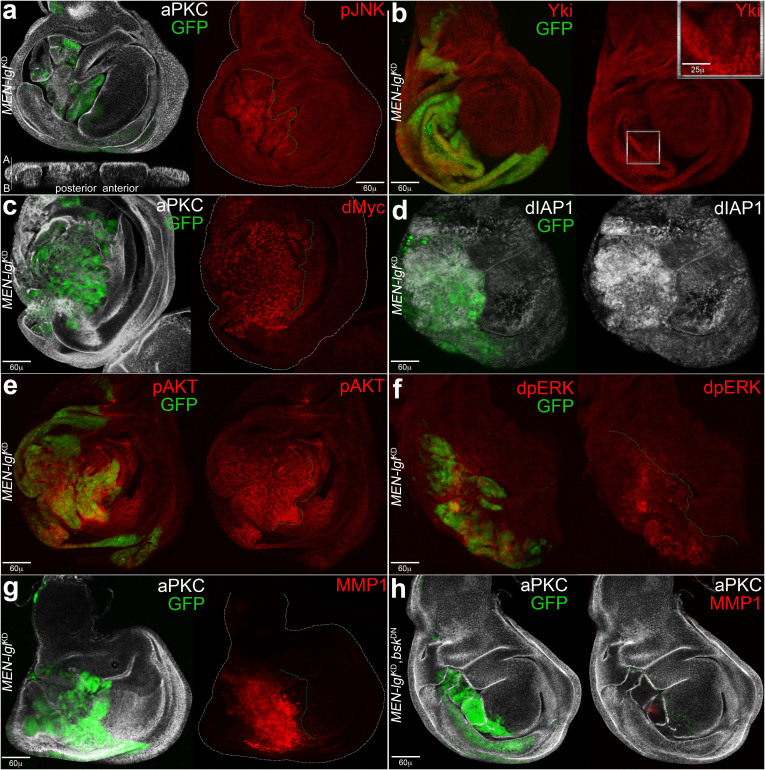
Several cancer-related pathways are implicated in the tumourigenic process triggered by the *MEN-lgl*^KD^ system. (a–g) Imaginal wing discs from *w*; *Rpl27A*^1^, *en-Gal4*, *UAS-GFP*/+; *UAS-lgl-RNAi*/+ individuals. (a) The overall disc structure is shown by aPKC staining; the posterior (P), GFP-positive compartment is visibly altered and the section along the *z* axis shows loss of apical-basal cell polarity. A: apical; B: basal. Ectopic expression of pJNK is shown in red. (b) Yki accumulation is visible in the P compartment (magnification). (c–g) Ectopic expression of dMyc, dIAP1, pAKT, dpERK and MMP1 respectively is evident in the P compartment. (h) Imaginal wing discs from *w, UAS-bsk*^DN^; *Rpl27A*^1^, *en-Gal4*, *UAS-GFP*/+; *UAS-lgl-RNAi*/+ individuals. As can be seen, disc structure, growth and invasiveness (MMP1 staining, red) are almost entirely rescued. The A/P border is dotted in green and disc contour is outlined in white. The genotype of the P compartment is indicated in each image.

*lgl* knockdown (*lgl*^KD^) has previously been used to induce tumour growth in the posterior (P) compartment of the wing disc under the control of the *engrailed* (*en*) promoter^22^ through the UAS-Gal4 binary system^34^. In that case, the *UAS-lgl*-hairpin transgene was used in conjunction with *UAS-dcr2* to enhance the effectiveness of RNAi^35^. The authors found that the neoplastic phenotype induced by *lgl*^KD^ was associated with deregulation of the Hpo pathway and JNK activation, and blocking the JNK signalling was sufficient to rescue both Hpo pathway deregulation and tumour growth^22^. In our hands, the *en>lgl-RNAi*-*dcr2* system did not induce overgrowth at 25°C. At 29°C morphological alterations of the P comparment were evident, but also the *en>dcr2* animals presented fragmentation of the P compartment and migratory behaviours of the P cells (not shown). We thus decided to use an alternative method to induce *lgl*^KD^-dependent neoplastic growth, that is a *Minute* background. *Minute* (*M*) are a group of dominant mutations in various ribosomal protein genes conferring a growth defect, so that *M*^+/−^ flies are of normal size but their development is delayed with respect to wild-type flies^36^. We chose a mutation in the *M(2)24F locus*, which encodes the Rpl27A protein, that we successfully used in a previous study^5^. Pupariation of the *w: Rpl27A*^1^*, en-Gal4, UAS-GFP/In(2LR)Gla, Bc* strain, hereafter referred to as *MEN* (from *Minute ENgrailed*), occurred at 6,5 days After Egg Laying (AEL) at 25°C, as opposed to the 5 days of a wild-type fly. When *MEN* flies were crossed to *UAS-lglRNAi* flies, hereafter referred to as *lgl*^KD^, the resulting progeny (*MEN-lgl*^KD^) showed a complete depletion of the Lgl protein in the posterior cells of the wing disc (Supplementary Fig. 1a) and a larval life lasting for about 11 days AEL at the end of which it died with no signs of differentiation. Figure 1 describes the main molecular features of the *MEN-lgl*^KD ^cells: the GFP^+^ P compartment appeared highly disorganised due to the loss of apical-basal cell polarity, as suggested by the *z* section in which the distribution of the apical marker aPKC is altered (Fig. 1a); in addition, P cells showed high levels of phosphorylated JNK (pJNK), indicative of pathway activation (Fig. 1a). Yki, the downstream effector of the Hpo pathway^17^, accumulated in many P cells (Fig. 1b, magnification), and its targets dMyc^37^,^38^ and dIAP1^17^ resulted consistently overexpressed (Fig. 1c, d). Ras signalling was also activated in our system, as can be inferred by the ectopic expression in the P compartment of its main effectors phosphorylated AKT (pAKT, Fig. 1e) and di-phosphorylated ERK (dpERK, Fig. 1f)^7^. The poor correlation between GFP-expressing cells and the nuclear markers analysed in Fig. 1b and 1f is frequently observed in transformed imaginal discs^22^ due to a rapid fluorescence decay in some tumour cells. Another recurrent trait of frank malignancies is the capability of cancer cells to degrade the basement mebrane (BM), and, as can be seen in Fig. 1g, the BM-degrading enzyme MMP1^21^ was strongly upregulated in the P cells. Finally, tumourigenesis in this genetic system was found to be JNK-dependent, as the expression of a dominant-negative form of *basket*, the gene encoding the JNK terminal kinase, rescued tumour growth (Fig. 1h). *w*/*w*,*UAS-bsk*^DN^;*Rpl27A*^1^,*en-Gal4*,*UAS-GFP*/+;*UAS-lgl-RNAi*/+ individuals differentiated into adult pharates; a small proportion of the pupae (15%, n = 335) eclosed into adults with no evident phenotypic alterations. To exclude non-specific effects due to saturation of the RNAi machinery, we repeated the same stainings on *MEN* wing discs in which the GFP expression was knocked down by a shRNA construct and, as can be seen in Supplementary Fig. 2, all the markers analysed showed a wild-type pattern (see figure legend for details).

Altogether, these data demonstrate that the *MEN-lgl*^KD^ system induces cancer growth through the same pathways as those found in previous studies utilising *lgl*^KD^^22^ or *lgl* mutations^5^, making it suitable for the investigation of multiple cancer-associated traits.

### *MEN-lgl*^KD^ tumours show a conserved response to hypoxic stress

**Figure 2:**
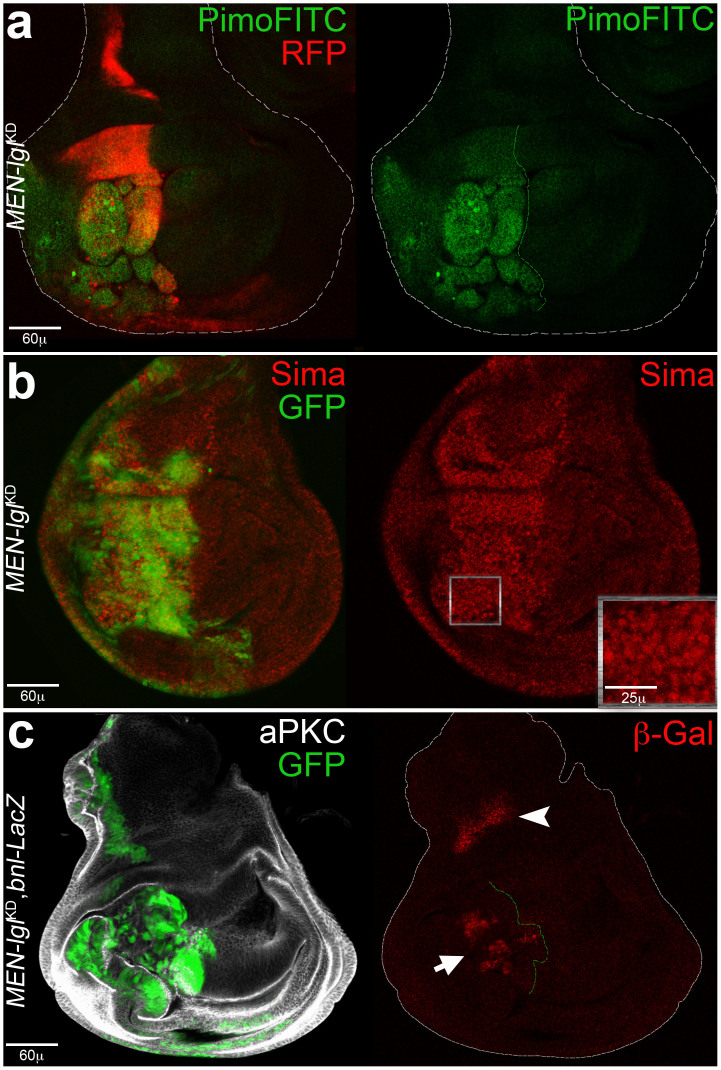
*MEN-lgl*^KD^ tumours show a response to hypoxic stress. (a) Imaginal wing discs from *w*; *Rpl27A*^1^, *en-Gal4*, *UAS-RFP*/+; *UAS-lgl-RNAi*/+ individuals show a hypoxic P compartment as indicated by positivity to the hypoxia probe pimonidazole (green). (b, c) Imaginal wing discs from *w*; *Rpl27A*^1^, *en-Gal4*, *UAS-GFP*/+; *UAS-lgl-RNAi*/+ individuals. (b) Sima/HIF1α accumulation is visible in the P compartment (magnification). (c): imaginal wing discs from *w*; *Rpl27A*^1^, *en-Gal4*, *UAS-RFP*/+; *UAS-lgl-RNAi*/*bnl-LacZ* individuals show that the *bnl*/*FGF* promoter is ectopically activated in many tumour cells (arrow); the physiological source of Bnl/FGF is indicated by the arrowhead. The A/P border is dotted in green and disc contour is outlined in white in a and c. The genotype of the P compartment is indicated in each image.

To check for oxygen shortage in growing cancers we took advantage of Pimonidazole, a chemical widely used for detection of hypoxia in mammalian tissues^39^. As can be seen in Fig. 2a, the FITC-conjugated anti-pimonidazole antibody (see Methods for details) specifically stained the P compartment (RFP^+^ cells) of *MEN-lgl*^KD^ wing discs. In the hypoxic environment, the *Drosophila* orthologue of the mammalian HIF1α, Similar (Sima), accumulated in the P cells (Fig. 2b, magnification), where it likely contributed to the activation of the *bnl*/*FGF* promoter (Fig. 2c, arrow). As the PI3K pathway is also implicated in Sima/HIF1α nuclear localisation^40^, the active pAKT signal (Fig. 1e) may also contribute to its accumulation. As expected, the control *MEN*-*GFP*^KD^ individuals did not show any signs of hypoxia (Supplementary Fig. 3a). Supplementary Fig. 1b shows the physiological source of Bnl/FGF in *MEN* wing discs (arrowhead), that was also found unaltered in *MEN-lgl*^KD^ samples as it does not fall within the *engrailed* domain (Fig. 1c, arrowhead). Ras is required for FGF signalling during development^41^ and, since its main downstream effectors appeared upregulated in our system (Fig. 1e, f), we knocked it down through RNAi in *MEN-lgl*^KD^ animals. This however resulted in growth inhibition of the P compartment and production of small wing discs (data not shown), thus it was not possible in this context to analyse the role of Ras signalling in FGF-dependent phenotypes.

These results show that *Drosophila* tumour cells carry out a hypoxic response that involves the same molecules as those found in mammalian cancers.

### *MEN-lgl*^KD^ tumours show migratory and tracheogenic behaviours

**Figure 3:**
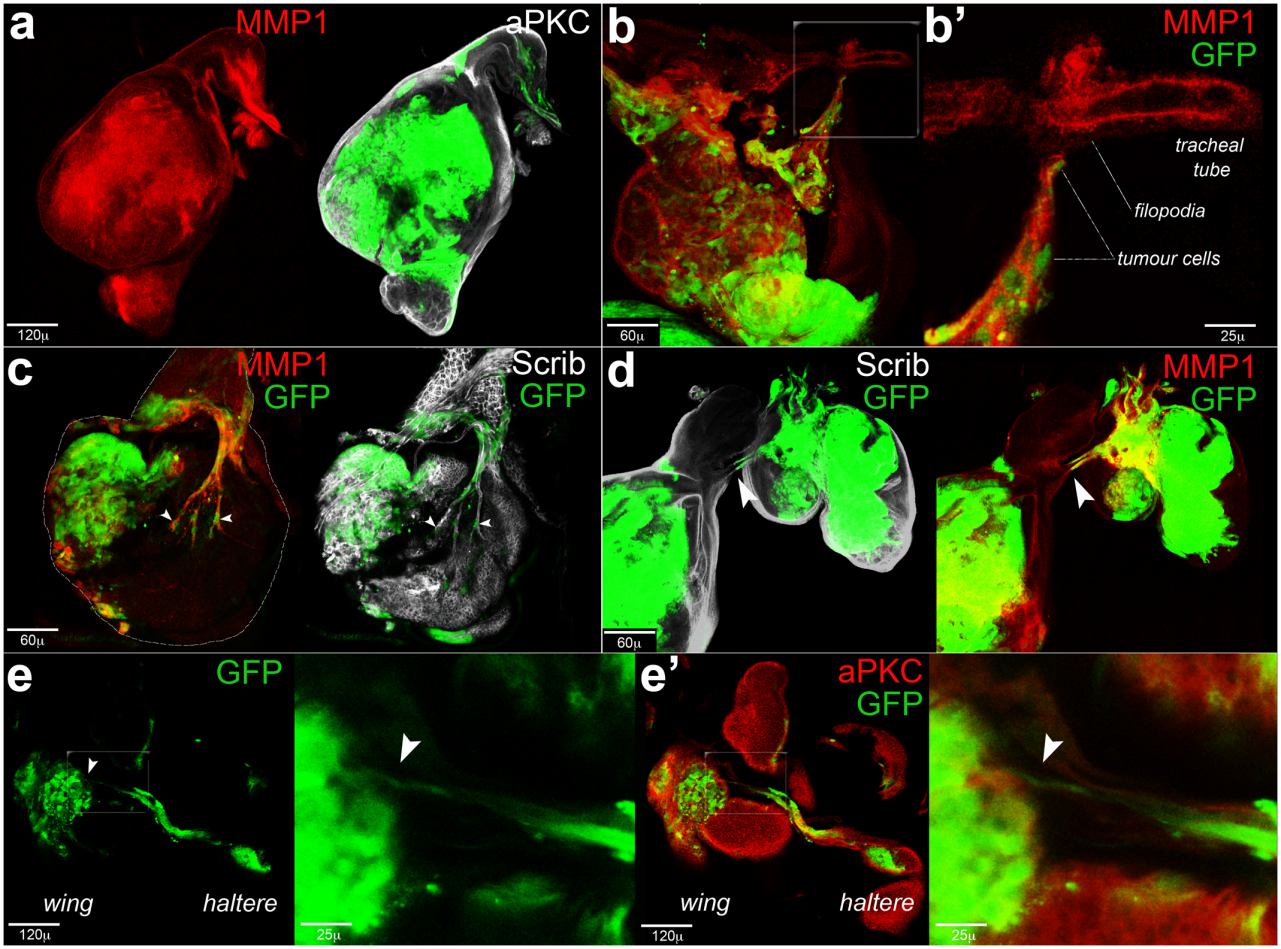
Migratory and tracheogenic behaviours of *MEN-lgl*^KD^ cells. Imaginal thoracic discs from *w*; *Rpl27A*^1^, *en-Gal4*, *UAS-GFP*/+; *UAS-lgl-RNAi*/+ individuals. (a) Imaginal wing discs from individuals at the extreme end of the larval life. As can be seen, the disc is nearly entirely composed of GFP-positive, highly invasive cells (MMP1 staining, red). (b, b′) An imaginal wing disc is shown in which some tumour cells migrate towards a tracheal tube and contact its filopodia. Tumour cells, filopodia and tracheal tube are indicated in the higher magnification in c. (c) An imaginal wing disc is shown in which tumour cells compose new tracheal branches. Some tumour, GFP-positive cells are indicated by the arrowheads. (d) Tracheal fusion in the thoracic triad (from left to right: wing disc, leg 3 disc and haltere disc). The arrowhead points out GFP-positive cells emerging from the haltere/leg discs fusing with the nearby wing tracheal tube. (e, e′) Haltere-to-wing trans-migration. GFP-positive cells coming from the haltere disc migrate towards the P compartment of the wing disc and enter tumour mass (arrowhead). This phenomenon can be observed in detail in Movie 1. In a and d, disc contour is outlined in white.

During cancer progression in the *MEN-lgl*^KD^ system, GFP^+^ cells escaped developmental constraints, trespassed the A/P boundary and colonised the entire disc. Figure 3a represents a wing disc from an individual at the extreme end of the larval life with diffuse MMP1 expression, a mark of high invasive potential. As MMP1 expression is also required for tracheal remodelling^28^, from this figure on it will also be used to identify neo-tracheal structures in alternative to/together with junctional proteins. As can be seen in Fig. 3b, at this stage cancer cells (GFP^+^) became highly migratory. Figure 3b′ zooms on a cluster of tumour cells engaged in a directional migration towards a tracheal tube, whose cells project several filopodia in the direction of the incoming cancer cells. As a control, the tracheal structure associated with control *MEN* wing discs is shown in Supplementary Fig. 1c (arrow). As described in the Introduction, mammalian cancer cells have been reported to participate in the formation of new vessels or to form vascular tubes on their own, a phenomenon called vascular mimicry (VM)^32^. We observed an analogous phenomenon: as can be noticed in Fig. 3c, tumour cells (GFP^+^) could form long branches; the arrowheads indicate GFP-positive nuclei of the cells forming the ectopic structures. Another mechanism correlated to oxygen deprivation in mammalian cancers is vascular co-option (VC), which begins with the migration of cancer cells along a vascular scaffold; as cancer growth proceeds, the vessel results completely embedded in the tumour mass, supplying it with oxygen^31^. As can be seen in Fig. 3d, GFP-positive cells could migrate along the tracheal vessels and fuse them to those of nearby structures; in this case, cancer cells coming from the haltere/leg 3 discs migrated along a tracheal vessel that appeared continuous with that of the wing disc (arrowhead). Finally, we report a phenomenon we named “trans-migration”, that is the emission of GFP^+^ branched structures by one disc of the thoracic triad towards the GFP^+^ compartment of a nearby disc. Figures 3e and 3e′ show the final step of a haltere-to-wing trans-migration, with GFP-positive cells coming from the haltere disc entering the P compartment of the wing disc. The whole sequence is shown in Supplementary Movie 1, where arrowheads indicate GFP-positive cells coming out of the haltere disc (arrow 1), migrating along the tracheal scaffold (arrow 2) and entering the P compartment of the wing disc (arrow 3). The biological significance of this phenomenon has not been addressed, but we speculate that it may represent a rapid way to deliver oxygen to a fast-growing tissue, with cancer cells moving towards an ectopic FGF source such that of the transformed P compartment of the wing disc (Fig. 2c). In principle, each cellular mass growing beyond a certain size should suffer from oxygen shortage, hence we also analysed *l(2)gl*^4^ mutant wing discs, that during larval development reach considerable dimensions^4^. As illustrated in Supplementary Fig. 4a, a large fraction of the mutant cells showed *bnl*/*FGF* promoter activation. In response to this, the tracheal network showed several ectopic branches visible at both the basal (Supplementary Fig. 4b) and the apical (Supplementary Fig. 4d) sides of the disc. Tracheal vessels were also included in the disc itself (Supplementary Fig. 4c). From all these data it appears evident that *Drosophila* tumour cells undertake a series of mechanisms in response to oxygen starvation that closely resemble those reported in mammals.

### *l(2)gl*^4^*Ras*^V12^ tumour cells suffer from hypoxia, express Bnl/FGF and form branched neo-structures

**Figure 4:**
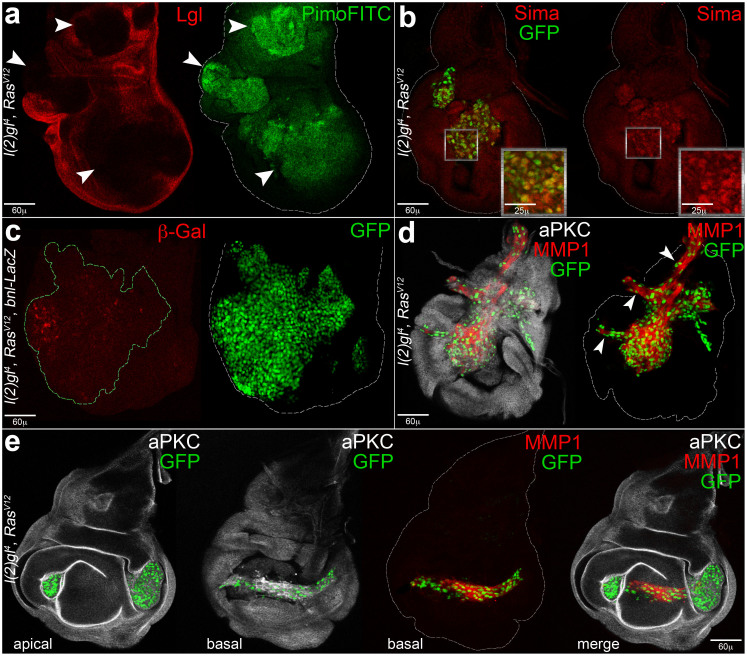
*l(2)gl*^4^*Ras*^V12^ cells respond to hypoxic stress through Bnl/FGF expression and formation of tubular neo-structures. (a, b) and (d, e) Imaginal wing discs bearing *l(2)gl*^4^*Ras*^V12^ clones induced in *yw*, *hs-Flp, tub-Gal4*/+; *tub-Gal80, FRT40A*/*l(2)gl*^4^, *FRT40A*; *UAS-Ras^V12^*/+ (a) and *yw*, *hs-Flp, tub-Gal4, UAS-GFP*/+; *tub-Gal80, FRT40A*/*l(2)gl*^4^, *FRT40A*; *UAS-Ras^V12^*/+ (b, d, e) individuals. (a) Imaginal wing discs in which clones are marked by the lack of Lgl protein (red^−^); as can be seen, mutant clones are positive for the pimonidazole staining (green) and thus hypoxic. (b) Most of the GFP^+^ cells show nuclear staining for Sima/HIF1αmagnification. (c) Imaginal wing discs bearing *l(2)gl*^4^*Ras*^V12^ clones induced in *yw*, *hs-Flp, tub-Gal4*/+; *tub-Gal80, FRT40A*/*l(2)gl*^4^, *FRT40A*; *UAS-Ras^V12^*/*bnl*-*LacZ* individuals. The large clone (GFP^+^) displays many cells activating *bnl*/*FGF* promoter. (d) The basal side of a disc is shown in which GFP^+^ tumour cells compose a large structure displaying several ramifications (arrowheads). (e) A disc showing two distinct clones at the apical side which appear to connect each other at the basal side by a tubule-shaped bridge formed by GFP^+^ cells; a merged image is also provided showing the position of the bridge relative to the apical clonal areas. In b and c, clonal area is dotted in green. In b-e, disc contour is outlined in white. Clone genotype is indicated in each image.

The *MEN-lgl*^KD^ system has proven valuable in the characterisation of the impressive tracheogenic processes that cancer cells carry out following hypoxic stress. However, cancer is by definition a clonal disease, and before proceeding with further analyses we shifted to a cooperative system, widely used to model clonal carcinogenesis, in which the *l(2)gl*^4^ loss-of-function mutation is combined with the oncogenic form of *Ras*, *Ras*^V12^^9^,^42^,^43^. We first confirmed the data collected in the previous system: as can be appreciated in Fig. 4a, the pimonidazole assay gave a strong signal in the clonal areas (marked by the absence of Lgl, arrowheads). As a control, Supplementary Fig. 3 shows the results of the same assay performed on *l(2)gl*^4^(Supplementary Fig. 3b) and *Ras*^V12^ (Supplementary Fig. 3c) clones; as can be seen, in both cases the pimonidazole assay failed to detect hypoxic cells. Figure 4b shows nuclear accumulation of Sima/HIF1α inside the clones (GFP^+^, see magnification), and many cells within the GFP^+^ clone shown in Fig. 4c activated the *bnl*/*FGF* promoter. The endogenous FGF source in the wild-type wing disc is shown in Supplementary Fig. 1d (arrowhead). Figure 4d illustrates the evident ectopic structures formed by GFP^+^ cells at the basal side of the disc; arrowheads point to branches departing from the central tumour mass. The endogenous tracheal network of a wild-type wing disc is shown for comparison in Supplementary Fig. 1e. In some cases, we observed tube-shaped structures composed of GFP^+^ cells whose morphology is shown with greater detail in Supplementary Fig. 5, in which several branches depart from a central mass. One of these branches, as can be seen in the *z* section, seems to enclose a rudimental lumen (Supplementary Fig. 5b–b″). In addition, it is worthwhile to mention the phenotype described in Fig. 4e: *l(2)gl*^4^*Ras*^V12^ clones that appeared isolated at the apical side of the disc were frequently found to be interconnected at the basal side by a bridge of tumour cells (GFP^+^). If such formations have any role in oxygen transport or in clonal expansion is currently unknown. To confirm a causative role for Bnl/FGF expression in tracheogenesis, we downregulated it by RNAi in the *l(2)gl*^4^*Ras*^V12^ clones, but we were not able to obtain viable larvae; as clones are induced randomly in our system, Bnl/FGF deprivation in some body districts possibly impairs larval development. Altogether, these findings point out a clear conservation of the hypoxic response in several genetic systems and identify tracheogenesis as a novel cancer hallmark in *Drosophila*, considerably similar to mammalian cancer-associated angiogenesis.

### *l(2)gl*^4^*Ras*^V12^ cells express the tracheal marker Trachealess and exhibit behaviours akin to mammalian vascular co-option and vascular mimicry

**Figure 5:**
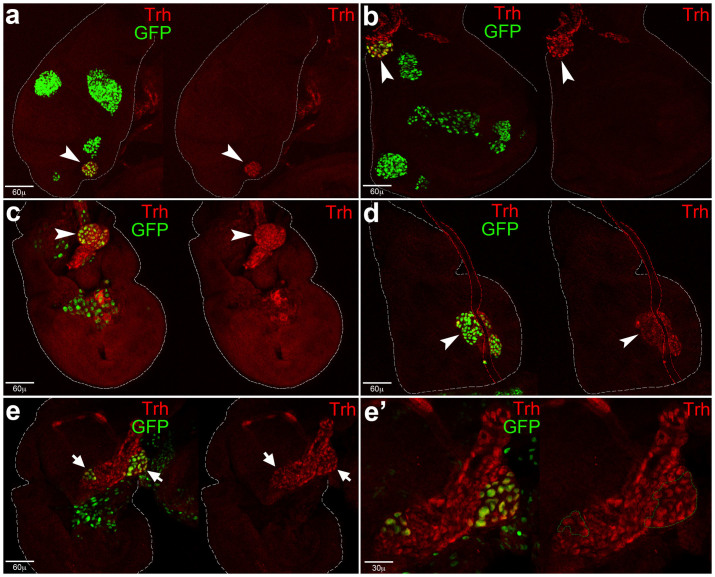
*l(2)gl*^4^*Ras*^V12^ cells expressing Trachealess grow attached to/wrapped around tracheal branches or help compose mosaic branches. (a–f) Imaginal wing discs bearing *l(2)gl*^4^*Ras*^V12^ clones induced in *yw*, *hs-Flp, tub-Gal4, UAS-GFP*/+; *tub-Gal80, FRT40A*/*l(2)gl*^4^, *FRT40A*; *UAS-Ras^V12^*/+ individuals. (a) A small GFP^+ ^clone expressing Trachealess (Trh) in the disc epithelium (arrowhead). (b) A GFP^+ ^clone growing attached to the wing tracheal branch (arrowhead). (c) Surface section of a GFP^+ ^clone (arrowhead) wrapped around the wing tracheal branch. (d) Cross-section of GFP^+ ^clones growing around (arrowhead) the transverse connective (outlined in red). (e) GFP^+ ^cells forming a mosaic tracheal branch (arrows) magnified in e′, where clones are dotted in green. Disc contours are outlined in white.

In about one-half of the wing discs where *l(2)gl*^4^*Ras*^V12^ clones were induced we observed that some GFP^+^ cells began to express the tracheal determinant Trachealess (Trh), known to participate in the activation of *btl/FGFR* transcription in several populations of cells during development^44^,^45^. These Trh-positive groups of cells were located randomly across the disc (Fig. 5a, arrowhead) but, more interestingly, they were often found close or connected to pre-existing tracheal branches (Fig. 5b, arrowhead) and, as in mammalian VC, wrapped around tracheal vessels (Fig. 5c, d, arrowheads). Trh-positive cells are restricted in the wild-type wing disc to the tracheal structures at the basal side of the disc (Supplementary Fig. 6a, the air sac and the transverse connective are labelled). Finally, *l(2)gl*^4^*Ras*^V12^ cells have also been found to compose mosaic branches together with resident tracheal cells (Fig. 5e, arrows), similar to mammalian cancer cells undergoing VM. Figure 5e′ shows a higher magnification of Fig. 5e. This evidence deserves deeper considerations. The fact that disc cells change fate upon transformation and are found in a different organ is reminiscent of a cancer stem cell behaviour^32^ but, before ascribing the observed phenotype to a trans-differentiation process, we must assure that the GFP-positive clones found inside the tracheal branches originated elsewhere in the disc. It is known from the literature that tracheal precursor cells proliferate until 5–7 h AEL, then formation of the larval tracheal network only occurs by cell migration and changes in cell size and shape^25^. In addition, at about 85 h AEL, some larval tracheoblasts begin to proliferate and form the air sac of the wing disc^41^. As clone induction accounts on mitotic recombination in our system, it can only occur in proliferating cells. We induced clones at about 48 h AEL, a time at which both embryonic and larval tracheoblasts are quiescent, and thus not prone to recombination. Supplementary Figure 6b shows a scheme describing the time frame for tracheoblast proliferation (green), clone induction (red) and tissue collection (blue) in wild-type individuals reared at 25°C. However, to exclude system pitfalls, we carried out a control experiment in which *l(2)gl*^4^*Ras*^V12^ clones were induced in the tracheal cells by substituting the ubiquitous *tub* promoter with the trachea-specific *btl/FGFR* promoter. To test the system, we induced clones at 0–4 hours AEL, and at the end of the larval life we obtained several individuals bearing tracheal clones; as an example, Supplementary Fig. 6c shows the anterior half of a late larva displaying many tracheal clones; the highlighted clone (arrow) is magnified in Supplementary Fig. 6d. Of note, the *l(2)gl*^4^and *Ras*^V12^ mutations were not able to induce overgrowth in the tracheal cells. We then used the same system to induce clones at 48 h AEL and no GFP-positive cells were observed in about 500 late larvae screened under a fluorescent stereoscope. To get into deeper details, we dissected 58 of these larvae and found no GFP-positive cells in the wing discs and connected tracheal network (Supplementary Fig. 6e). This was convincing evidence that the *l(2)gl*^4^*Ras*^V12^ cells growing in the tracheal walls shown in Fig. 5e, e′ are cells of the disc epithelium which, following malignant transformation, acquired a tracheal fate. Altogether, these results demonstrate that both VC and VM are conserved in *Drosophila* cancers, emphasising this model as a powerful tool in which to investigate the molecular basis of these still poorly charachterised mechanisms.

### The tracheal switch of *l(2)gl*^4^*Ras*^V12^ cells is coupled with changes in Polycomb and STAT expression

**Figure 6:**
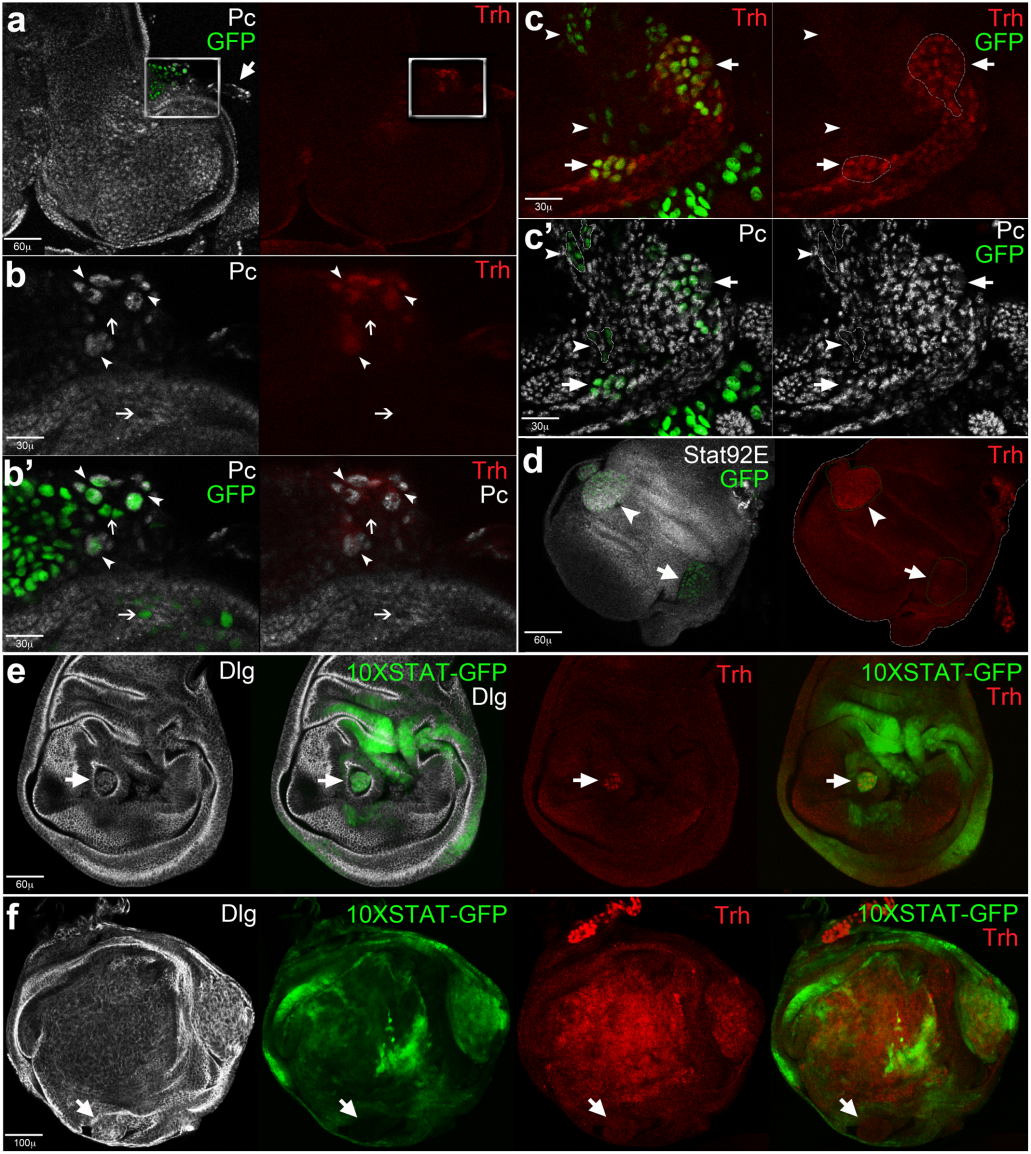
The tracheal switch of *l(2)gl*^4^*Ras*^V12^ cells is coupled with Polycomb modulation and STAT92E ectopic expression. (a–f) Imaginal wing discs bearing *l(2)gl*^4^*Ras*^V12^ clones induced in *yw*, *hs-Flp, tub-Gal4, UAS-GFP*/+; *tub-Gal80, FRT40A*/*l(2)gl*^4^, *FRT40A*; *UAS-Ras^V12^*/+ individuals. (a–b′) A GFP^+^ clone growing attached to a tracheal branch (a) shows Polycomb (Pc) expression in Trh-positive cells (b, arrowheads), while Pc expression is missing in GFP^+^, Thr-negative cells (b and b′, arrows). (c, c′) GFP^+^, Trh-positive cells (c, arrows) included in a mosaic tracheal ramification. Arrowheads indicate GFP^+^ cells that do not express Trh (c) and downregulate Pc (c′). (d) Two GFP^+^ clones in which Stat92E and Trh are coherently expressed; the arrowhead indicates a clone in which both proteins are present and the arrows points out a clone in which both proteins are missing. (e, f) Imaginal wing discs bearing *l(2)gl*^4^*Ras*^V12^ clones induced in *yw*, *hs-Flp, tub-Gal4*/+; *tub-Gal80, FRT40A*/*l(2)gl*^4^, *FRT40A*; *UAS-Ras^V12^*/*STAT-GFP* individuals. The mutant clones are marked by loss of cell polarity highlighted by Dlg staining. The arrow in e points to a clone positive for both STAT and Trh, while the arrow in f points to a clone negative for both STAT and Trh. Clones are dotted in white in c, c′ and in green in d.

In cultured imaginal discs, cellular plasticity was found to be tuned, among others, by proteins of the Polycomb group (PcG)^46^, known to maintain cellular fate by controlling the expression pattern of many developmental regulators^47^. With the aim to test if Trh-positive and Trh-negative *l(2)gl*^4^*Ras*^V12^ cells associated to the tracheal walls displayed any differences in Pc levels, we performed a double Pc-Trh staining. Figure 6a shows a wing disc in which the outlined GFP^+^ clone is growing attached to a tracheal tube (arrow), and part of its cells express Trh. A higher magnification of the region squared in Fig. 6a is shown in Fig. 6b, b′. As can be seen in Fig. 6b, all the cells expressing Trh showed high levels of Pc (arrowheads), while GFP^+^ cells lacking Trh expression downregulated Pc (compare Fig. 6b′ to Fig. 6b, arrows). Another example of this behaviour is shown in Fig. 6c, c′: GFP^+^ cells that did not express Trh (arrowheads) coherently showed low levels of Pc (outlined in Fig. 6c′). On the other hand, GFP^+^ cells expressing Trh (Fig. 6c, arrows and dashes) upregulated Pc (Fig. 6c′, arrows). Trh and Pc co-expression was confirmed by an analysis performed on *l(2)gl*^4^*Ras*^V12 ^clones located within or around a tracheal vessel, in which GFP^+^ cells were scored for the presence of both markers (Supplementary Fig. 7a). It thus seemed that *l(2)gl*^4^*Ras*^V12^ cells associated to a tracheal tube that did not acquire a tracheal fate remained in an unprogrammed state, marked by Pc downregulation. Possibly, Trh-expressing *l(2)gl*^4^*Ras*^V12^ cells acquired a tracheal fate upon exposure to hypoxia or to other local factors. In mammals, it is documented that the pro-angiogenic factors produced by hypoxic cancer cells can be induced by cooperation between HIF1α and STAT3, the JAK-STAT cascade downstream effector^48^. These molecules are indeed frequently co-upregulated in mammalian hypoxic cancers^49^ and have been recently associated with VM in gastric cancer^50^. In *Drosophila*, STAT92E is the only member of the STAT family^51^. The regulatory regions of several genes involved in tracheal development, including *trh*, have shown to contain specific enhancers for STAT92E^52^. As can be seen in Fig. 6d, a Trh-expressing clone (arrowhead) showed STAT92E staining, while the Trh-negative clone (arrow) did not. We also monitored STAT activity in *l(2)gl*^4^*Ras*^V12^ clones through a STAT-GFP reporter, whose activity in a wild-type disc is showed in Supplementary Fig. 7b. As can be seen in Fig. 6e, GFP and Trh are co-expressed in the same cells (arrow), while in Fig. 6f a wing disc is shown in which the small clone indicated by the arrow does not show STAT activation and does not express Trh. We then screened a number of *l(2)gl*^4^*Ras*^V12^ clones for the presence of both markers and, as can be seen in Supplementary Fig. 7c, both in the proximal and distal regions of the disc, while several STAT92E-positive clones do not show Trh expression, the most part of the Trh-positive clones are also STAT92E-positive. We then downregulated STAT92E by RNAi in *l(2)gl*^4^*Ras*^V12^ clones and observed that only 6% (n = 34) of the wing discs examined carried a GFP^+^, Trh-expressing clone, compared to the 50% (n = 23) observed in the control experiment. These data suggest that STAT92E may be involved in Trh ectopic expression in *l(2)gl*^4^*Ras*^V12^ cells, but additional work is required to confirm a direct implication of the JAK/STAT signalling in Trh regulation.

### Active JNK signalling regulates Polycomb and STAT92E expression in *l(2)gl*^4^*Ras*^V12^ cells

**Figure 7:**
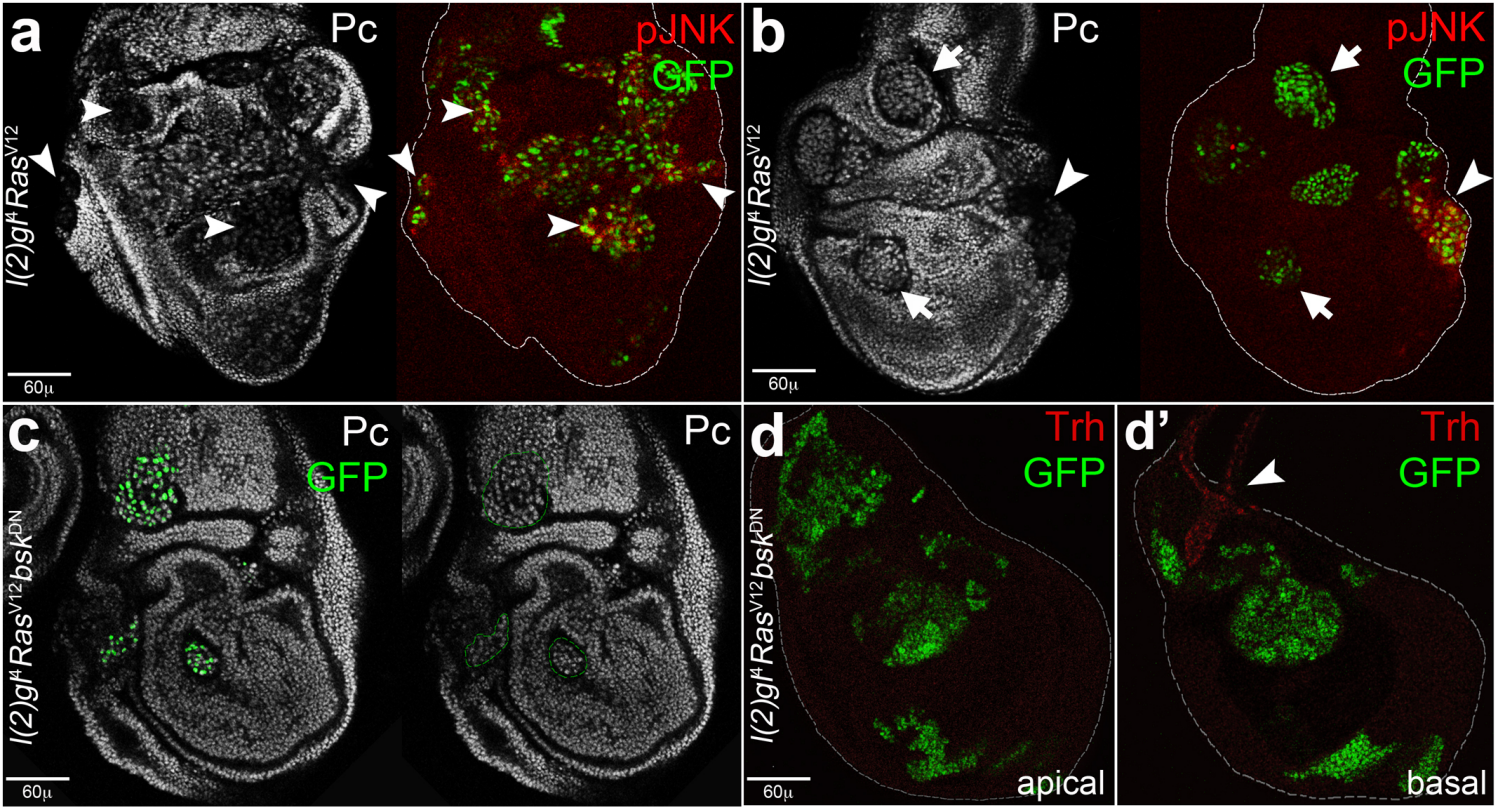
The chromatin remodelling protein Polycomb is downregulated in *l(2)gl*^4^*Ras*^V12^ clones showing an active JNK signalling. (a, b) imaginal wing discs bearing *l(2)gl*^4^*Ras*^V12^ clones induced in *yw*, *hs-Flp, tub-Gal4, UAS-GFP*/+; *tub-Gal80, FRT40A*/*l(2)gl*^4^, *FRT40A*; *UAS-Ras^V12^*/+ individuals. (a, b) The GFP^+^ clones showing ectopic expression of pJNK also present downregulated Pc (arrowheads). (c–d′) Imaginal wing discs bearing *l(2)gl*^4^*Ras*^V12^*bsk^DN^* clones induced in *yw*, *hs-Flp, tub-Gal4, UAS-GFP*/*UAS-bsk*^DN^; *tub-Gal80, FRT40A*/*l(2)gl*^4^, *FRT40A*; *UAS-Ras^V12^*/+ individuals. By silencing the JNK signalling cascade, Pc expression is rescued in *l(2)gl*^4^*Ras*^V12^ cells (c, clones dotted in green) and Trh expression is restricted to the normal tracheal structure (d′, arrowhead). In a, b and d disc contour is outlined in white. Clone genotype is indicated beside each image.

Previous work suggested that polarity-deficient cells forming tumours in the wing disc are dependent on JNK signalling for growth and invasiveness^20^,^21^,^22^. In the present work, tumourigenesis induced by the *MEN-lgl*^KD^ system was also found to be JNK-dependent (Fig. 1h). We thus checked JNK activation in the clonal system, and found that many *l(2)gl*^4^*Ras*^V12^ clones showed high pJNK staining (Fig. a, b). Since JNK signalling has been shown to specifically suppress the expression of Polycomb group proteins in *Drosophila* and mammalian cells^53^, we sought to find a correlation between the two and, as can be inferred by Fig. 7a, b, Pc expression appeared to be correlated to JNK activation. In particular, in Fig. 7a a disc is shown in which all *l(2)gl*^4^*Ras*^V12^ clones activated the JNK signalling and repressed Pc expression (arrowheads), and Fig. 7b shows a disc in which the only clone activating JNK signalling downregulated Pc (arrowhead). The remaining clones, possibly exposed to different combinations of local factors, did not show pJNK staining and expressed Pc (arrows). Strikingly, inhibition of the JNK cascade in *l(2)gl*^4^*Ras*^V12^ clones rescued Pc expression (Fig. 7c, outlined clones) and repressed STAT92E (data not shown) and Trh (Fig. 7d) ectopic expression; Trh staining resulted indeed restricted to the normal wing tracheal network (Fig. 7d′, arrowhead). Altogether, these findings suggest that some *l(2)gl*^4^*Ras*^V12^ cells activate JNK signalling that, in turn, represses Pc expression and activates STAT92E. It has been recently shown that Pc mutation triggers neoplastic growth in the eye imaginal tissue through derepression of the JAK-STAT ligand *loci*^54^; this suggests that STAT92E ectopic expression may be modulated by Pc protein levels also in our system.

## Discussion

**Figure 8:**
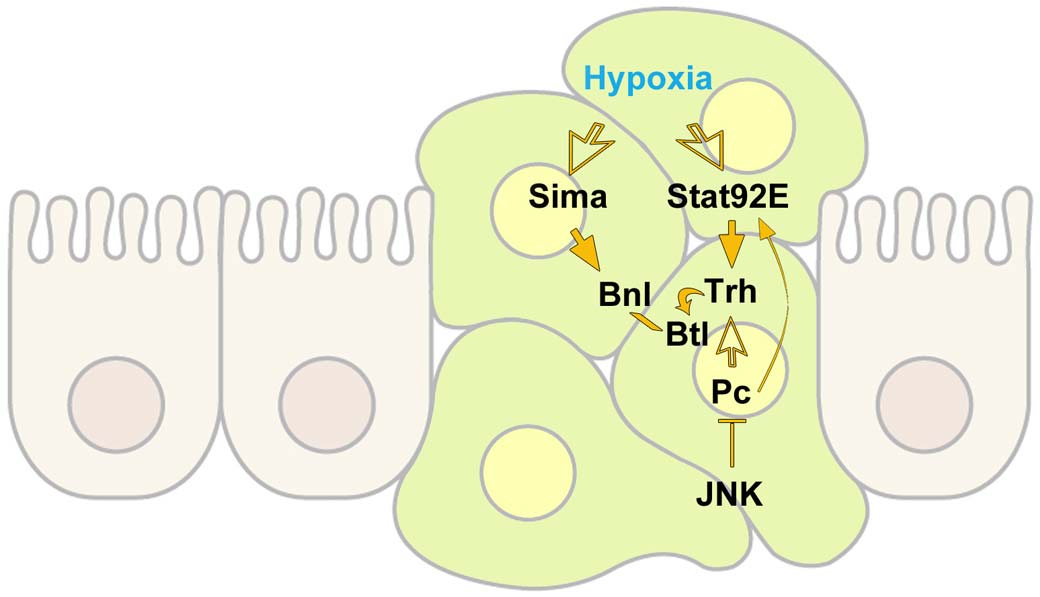
A model for the tracheal switch of *l(2)gl*^4^*Ras*^V12^ cells. The overgrowing clone at the centre of the figure (green) is surrounded by normal epithelial cells (hazel). Activated JNK downregulates Pc, thus derepressing STAT92E. Upon hypoxic stress, Sima/HIF1α is stabilised, enters nucleus and activates *bnl/FGF* transcription, while STAT92E-dependent Trh expression may, in turn, strengthen *btl/FGFR* promoter activation. All these events are likely to force undifferentiated cells to acquire a tracheal fate. The empty arrows represent cellular conditions that are permissive to the expression of the indicated molecules, whereas full arrows and bars represent a direct effect on the expression/activity of the indicated molecules.

Targeted therapy is the most thoughtful tool so far developed to challenge cancer disease, as it is based on a precise knowledge of the key mechanisms supporting cancer growth. Upon oxygen shortage, cancer cells are known to express pro-angiogenic factors such as VEGF and FGF and this, in turn, stimulates nearby vessels to form new branches to feed the expanding mass. Anti-VEGF molecules have thus been developed to counteract this phenomenon^55^. Unfortunately, cancer cells were found to adopt alternative strategies to access oxygen sources, such as the seizure of pre-existing vessels or the formation of a capillary-like network on their own, thus becoming resistant to anti-VEGF therapies. The molecular basis of these mechanisms, respectively known as vascular co-option (VC) and vascular mimicry (VM), is largely unknown^31^,^32^. Future therapies aimed at depriving cancer cells of oxygen might thus be most beneficial if based on multi-target approaches, and the identification of the molecular changes at the basis of each different mechanism of blood supply is of great interest. As *Drosophila* has an open circulatory system, it has long been considered unsuitable to study cancer-associated angiogenesis, and this trait was so far unexplored in the fly. However, oxygen is spread throughout the body by the tracheal system, an interconnected tubular network whose regulation is significantly analogue to that of mammalian vascular tree^25^. Furthermore, the functional analogies between the *Drosophila* tracheal system and the mammalian vascular tree may go well beyond oxygen transportation. In a pivotal study about cooperative oncogenesis, the authors clearly showed GFP^+^ cancer cells entrapped within a tracheal tube^9^, and a recent study found that midgut homeostasis is regulated in the adult fly by the Dpp/TGFβ supplied by the tracheal cells^56^. These data demonstrate that tracheal function, both in physiological and pathological conditions, is not restricted to air exchange. The tracheal system may also be involved in the production and/or transportation of growth factors acting locally or systemically for inter-organ communication, support cancer growth and vehiculate cancer cells during metastatic disease. Our analysis of the tracheal changes occurring throughout tumourigenesis in *Drosophila* larval epithelia showed that they are closely related to the vascular modifications seen in mammalian cancers. We were able to recognise ectopic tracheal sprouting in FGF-expressing cancer tissues (Supplementary Fig. 4), migration of cancer cells towards nearby tracheal tubes (Fig. 3b, b′), tracheal co-option (Fig. 5c, d) and tracheal mimicry (Fig. 3c and Fig. 4d), frequently associated with differentiation of cancer cells into tracheal cells (Fig. 5). Furthermore, these vascular alterations were supported by the same molecular mechanisms known to respond to hypoxic stress in mammalian cancers. Indeed, in *Drosophila* cancer cells suffering from oxygen deprivation the hypoxia-inducible factor Sima/HIF1α translocated into the nucleus (Fig. 4b) and the *bnl/FGF* promoter resulted activated (Fig. 2c and Fig. 4c). This, in turn, may trigger all the tracheogenic phenotypes above described. Cancer cells expressing the tracheal determinant Trachealess (Trh) were frequently found to compose mosaic branches together with resident tracheal cells (Fig. 5e, e′ and Fig. 6c, c′). VM may indeed lead to mosaic vessels that are characterised in mammals by alternating cancer and endothelial cells in the capillary walls^57^. We investigated the molecular basis of this phenomenon starting from the evidence that cellular plasticity underlying the differentiation process involves reprogramming of cell identity, mediated by local signals and by internal changes at the transcriptional and epigenetic levels. Cell identity is established and maintained in both *Drosophila* and mammals through the organisation of specific chromatin domains by the Polycomb (Pc) and Trithorax (Trx) group proteins^47^, hence we investigated Pc expression in transformed cells. We found that the undifferentiated state of most cancer cells was marked by Pc downregulation; upon transition from an undifferentiated to a tracheal fate, they started to express Pc again (Fig. 6b–c′ and Supplementary Fig. 7a). We also found that Trh ectopic signal correrelated with STAT92E expression/activity (Fig. 6d–f and Supplementary Fig. 7c), the effector of the JAK-STAT signalling cascade in *Drosophila*^51^. As described in the Results section, Trh ectopic expression was severely restricted upon STAT92E downregulation by RNAi. The expression of Pc group proteins has been shown to be specifically suppressed by the JNK cascade in *Drosophila* and mammalian cells^53^. We were able to show that these mechanisms are conserved during cancer growth, finding a correlation between active JNK signal and Pc repression (Fig. 7a, b). Pc expression was consistently rescued following JNK inhibition (Fig. 7c), as were STAT92E (data not shown) and Trh (Fig. 7d) ectopic signals. In support of our findings, STAT92E was found to be directly activated by JNK signalling in a similar tumour model^10^, and Pc mutation was known to trigger neoplastic growth in the eye imaginal tissue through derepression of the JAK-STAT ligand *loci*^54^. The tendency of malignant clones to grow around tracheal vessels has also been observed in metastatic tumours induced by combined expression of activated Src and Ras^58^. Such masses express Bnl/FGF and show abnormal tracheal vessels, suggesting they are formed *de novo* by tumour cells^58^. Ectopic tracheal vessels were also found in a *Drosophila* model of glioma^59^. These observations suggest that the mechanisms we report, summarised in Fig. 8, are not specific to the genetic systems or to the organs utilised in our study, but rather represent a general feature of cancer growth in *Drosophila*. The use of the fruitfly for future research on this topic will open new possibilities in the dissection of the genetic and molecular basis of angiogenic strategies in human cancers.

## Methods

### Fly stocks and husbandry

Fly stocks used in this study were from the Bloomington Stock Center (http://flystocks.bio.indiana.edu/) except for *w*; *UAS*-*lgl-*RNAi III (VDRC nr. 51249), *w*; *btl-RFPmoe* III (M. Affolter) and *w; 10xSTAT-GFP* (T. Vaccari and M. Crozatier). All the RNAi transgenes were validated by specific antibody staining or reporter constructs. Stocks and experimental crosses were all raised on standard medium at 25°C. For larval staging, eggs from 15–20 females were collected for 8 hours so to avoid developmental delays due to overcrowding, except for the experiment illustrated in Supplementary Fig. 6C where egg depositions lasted 4 hours.

### Mutant, *MEN*-*lgl*^KD^ and clonal analyses

For the experiment presented in Supplementary Fig. 4, *w*; *l(2)gl*^4^, *FRT40A*/*In(2LR)Gla, Bc*; *bnl*-*LacZ*/*TM6b* females were crossed to *w*; *l(2)gl*^4^, *FRT40A*/*In(2LR)Gla, Bc*; *btl-Gal4*, *UAS-mCD8::GFP*/*TM6b* males and *l(2)gl*^4^ homozygous larvae were grown up to 10 days AEL before dissection. For the *MEN-lgl*^KD^ analysis, *w; Rpl27A*^1^*, en-Gal4, UAS-GFP/In(2LR)Gla, Bc* females were crossed to *w; UAS-lgl-RNAi* males and larvae were grown for 8 days AEL (Figs. 1, 2, 3c, e, e′, Supplementary Fig. 1a and Supplementary Movie 1) or 11 days AEL (Fig. 3a–c′ and d) before tissue isolation. For the *MEN* control experiments (Supplementary Fig. 1b, c), *w; Rpl27A*^1^*, en-Gal4, UAS-GFP/In(2LR)Gla, Bc* larvae were grown up to 6 days AEL before dissection. For the *MEN-GFP^KD^* control experiments *w; Rpl27A*^1^*, en-Gal4, UAS-GFP/UAS-GFP-RNAi* larvae were grown up to 6 days AEL before dissection (Supplementary Fig. 2 and Supplementary Fig. 3a). For mosaic analysis, the MARCM system was used^60^. Larvae were heat shocked at 48 ± 4 hours AEL for 20 minutes at 37°C in a circulating water bath; for the control experiment illustrated in Supplementary Fig. 6c, embryos were heat shocked immediately after the 4-hours deposition. Larval tissues were collected and treated at 110–120 hours AEL.

### Immunohistochemistry

Discs were dissected in PBS paying attention to preserve the local tracheal structures, fixed in 3,7% formaldehyde in PBS, washed in 0.3%Triton in PBS and mounted in Fluoromount-G (Southern Biotechnology Associates, Inc.). The following primary antibodies were used: rabbit anti-aPKCζ (1:200, sc-216, Santa Cruz Biotechnology, Inc., Santa Cruz, CA, USA); mouse anti-phospho-JNK (1:400, clone G9, Cell Signaling Technology Inc., Danvers, MA, USA); rabbit anti-Yorkie (1:400, K. D. Irvine); rabbit anti-phosphoAKT (1:200, Ser505, Cell Signaling Technology Inc., Danvers, MA, USA); mouse anti-dpERK (1:200; Sigma); mouse anti-dMyc (1:5, P. Bellosta); mouse anti-dIAP1 (1:100, B. A. Hay); rabbit anti-MMP1 (1:50, 3A6B4, DSHB, University of Iowa, Iowa City, IA, USA); mouse Hypoxyprobe FITC-conjugated (1:200, Hypoxyprobe); mouse anti-β-Gal (1:20, 40-1a, DSHB, University of Iowa, Iowa City, IA, USA); rabbit anti-Lgl (1:500, D. Strand); mouse anti-Dlg (1:50, 4F3, DSHB, University of Iowa, Iowa City, IA, USA); rabbit anti-Scrib (1:100, C. Q. Doe); rabbit anti-Trh (1:20; J. Casanova); rat anti-Trh (1:200; L. Jiang); rabbit anti-Pc (1:500, sc-25762- Santa Cruz Biotechnology, Inc., Santa Cruz, CA, USA); rabbit anti-STAT92E (1:200, S. X. Hou); rabbit anti-Sima: a polyclonal serum was produced for this study by PRIMM SRL. Rabbits were immunised with the antigenic peptide NH2-TYVDDKMHDLLGYIC-COOH, aa. 332–344 of the Similar protein sequence. Rabbit 2 gave the expected signal in both WB and IF performed on *tub>sima* larvae pre-treated with 300μM CoCl_2_ for 24 hours to induce Sima stabilisation. Non pre-treated *tub>sima* larvae were used as a control. Secondary antibodies were: Alexa Fluor 555 goat anti-mouse and anti-rabbit (Invitrogen Corporation, Carlsbad, CA, USA) and DyLight 649 goat anti-mouse and anti-rabbit (Jackson ImmunoResearch Laboratories, Inc., West Grove, PA, USA).

### Image analysis

Fluorescent images were taken on a Leica TCS SP2 confocal microscope and entire images were processed with Adobe Photoshop software; images are all from a single *xy* stack. ImageJ free software from NIH, Bethesda, MD, USA was used to rebuild the projections along the *z* axis starting from 35–50 *xy* stacks. Unless otherwise specified, each experiment is based on the observation of 55–80 wing discs, and all figures show a phenotype found in over 75% of the samples, that we consider highly representative.

### Pimonidazole conjugation assay

To check for hypoxic tissues, larval carcasses were exposed to a 400μM solution of a bio-reactive drug, Pimonidazole, for 45 minutes in Gibco Schneider's medium prior to regular fixation (http://site.hypoxyprobe.com/knowledge-center-articles/HP-1-Kit-Insert.pdf). This drug forms adducts with thiol-containing proteins at pO_2_ ≤ 10 mm Hg. Carcasses were then washed in PBS and fixed in 3,7% formaldehyde in PBS. A mouse-FITC-Mab against was added to detect the pimonidazole adducts.

## Author Contributions

D.G. and A.P. conceived the study and designed the experiments; M.S., E.F. and F.F. performed the experiments; D.G. and A.P. analysed the data and wrote the paper.

## Supplementary Material

Supplementary Information Supplementary Information

Supplementary Information Supplementary movie 1
