# Supplementary material for: Multiple strategies of oxygen supply in Drosophila malignancies identify tracheogenesis as a novel cancer hallmark
Source: Sci Rep. 2015 Mar 12;5:9061. doi: 10.1038/srep09061 (PMC4357021; doi:10.1038/srep09061)
Supplement: Supplementary Information [file srep09061-s1.pdf]

Multiple strategies of oxygen supply in *Drosophila* malignancies identify tracheogenesis as a novel cancer hallmark

Daniela Grifoni*, Manuela Sollazzo, Elisabetta Fontana, Francesca Froldi# and Annalisa Pession

Department of "Farmacia e Biotecnologie", University of Bologna, Bologna, Italy

# Present address: Peter MacCallum Cancer Centre, Melbourne, Australia

*Corresponding author

Running title: Tracheogenesis in *Drosophila* malignant tumours

Corresponding author:

Daniela Grifoni, PhD

Department of "Farmacia e Biotecnologie"

University of Bologna

Via Selmi 3, 40126 Bologna, Italy

Telephone number +39(0)512094179

Fax number +39(0)512094286

E-mail address: daniela.grifoni@unibo.it

Web URL http://www.unibo.it/Faculty/default.htm?UPN=daniela.grifoni%40unibo.it

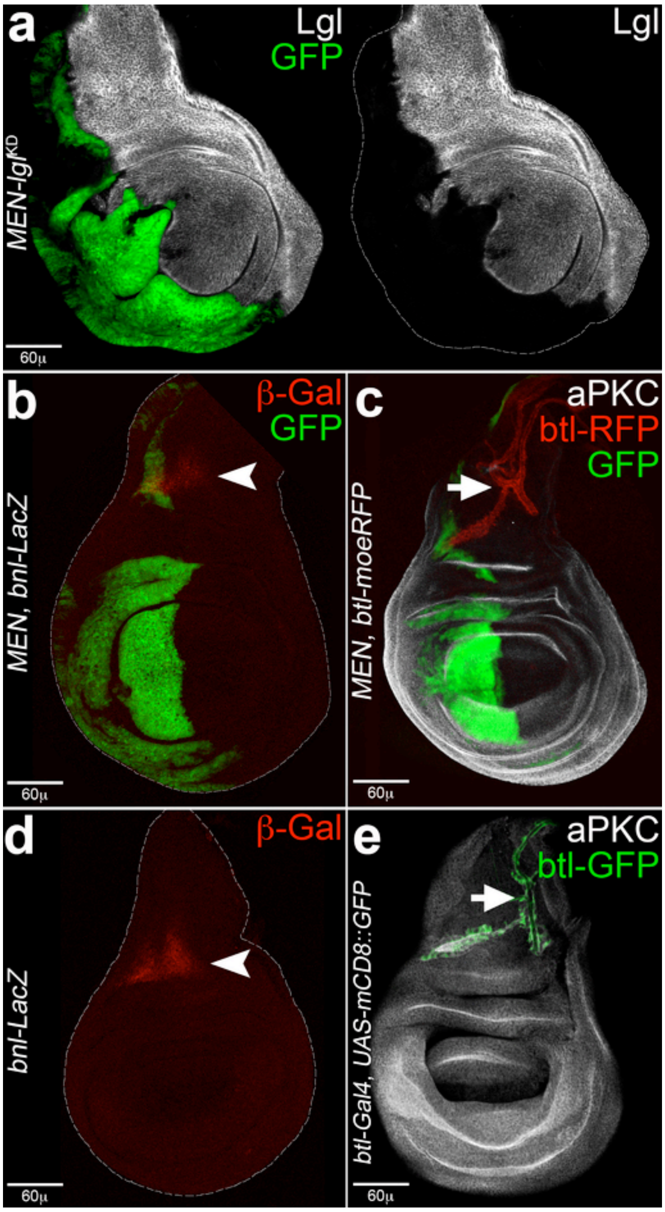

Supplementary Figure 1 | Bnl/FGF and Btl/FGFR patterns in *MEN* and wild-type discs. (**a**) Imaginal wing discs from *w*; *Rpl27A*^{1}, *en-Gal4*, *UAS-GFP*/+; *UAS-lgl-RNAi*/+ individuals. Lgl staining shows complete absence of the protein in the P compartment (GFP^{+}). (**b**) Imaginal wing discs from *w*; *Rpl27A*^{1}, *en-Gal4*, *UAS-GFP*/+; *bnl-LacZ*/+ individuals; the *bnl* reporter reveals the source of Bnl/FGF in the *notum* region of the disc epithelium (arrowhead). (**c**) Imaginal wing discs from *w*; *Rpl27A*^{1}, *en-Gal4*, *UAS-GFP*/+; *btl-RFPmoe*/+ individuals; the *btl* reporter highlights the tracheal structures connected to the basal side of the wing disc (arrow). (**d**) Imaginal wing discs from *w*; *bnl-LacZ*/*TM6b* individuals; the *bnl* reporter reveals the wild-type source of Bnl/FGF in the disc epithelium (arrowhead). (**e**) Imaginal wing discs from *w*; *btl-Gal4, UAS-mCD8::GFP* individuals; the *btl* reporter highlights the tracheal structures contacting the basal side of the wing disc (arrow). Disc contour is outlined in white in **a**, **b** and **d**. Genotypes are indicated in each image.

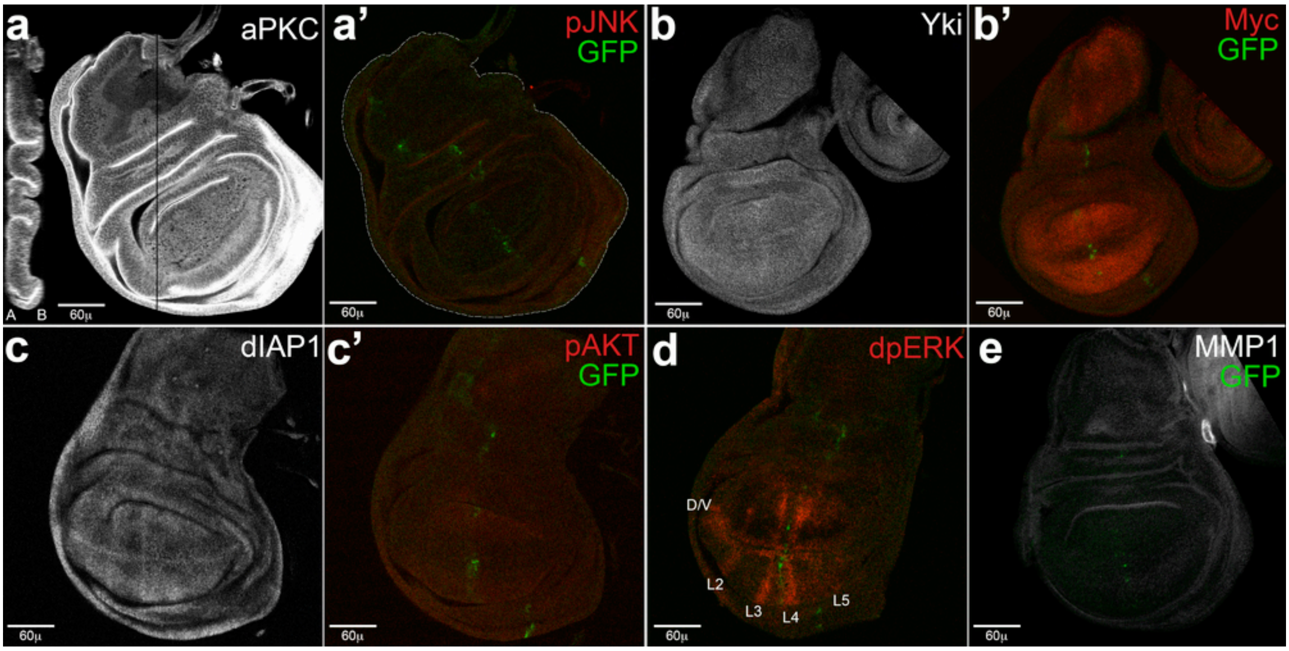

Supplementary Figure 2 | Disc morphology and tumour-associated markers are not altered in the *MEN-GFP*^{KD} control system.

(**a**-**e**) Imaginal wing discs from *w*; *Rpl27A*^{1}, *en-Gal4*, *UAS-GFP*/+; *UAS-GFP-RNAi*/+ individuals. (**a**-**a'**) The posterior (P) compartment is not altered: the section along the *z* axis shows a correct apical- (A) basal (B) cell polarity and the JNK cascade is not activated. (**b**-**b'**) Yki staining is uniform across the disc and the Myc pattern is wild-type (ref. 8). (**c**-**c'**) dIAP1 staining is uniform across the disc and AKT is not activated. (**d**) dpERK pattern is restricted to the future veins as it is in the wild-type disc (DV=dorsal-ventral margin; L2-5=Longitudinal veins 2-5). (**e**) MMP1 activity is absent. The P compartment (to the left) is identified thanks to a residual GFP fluorescence at the A/P border, and disc contour is outlined in white in **a'**.

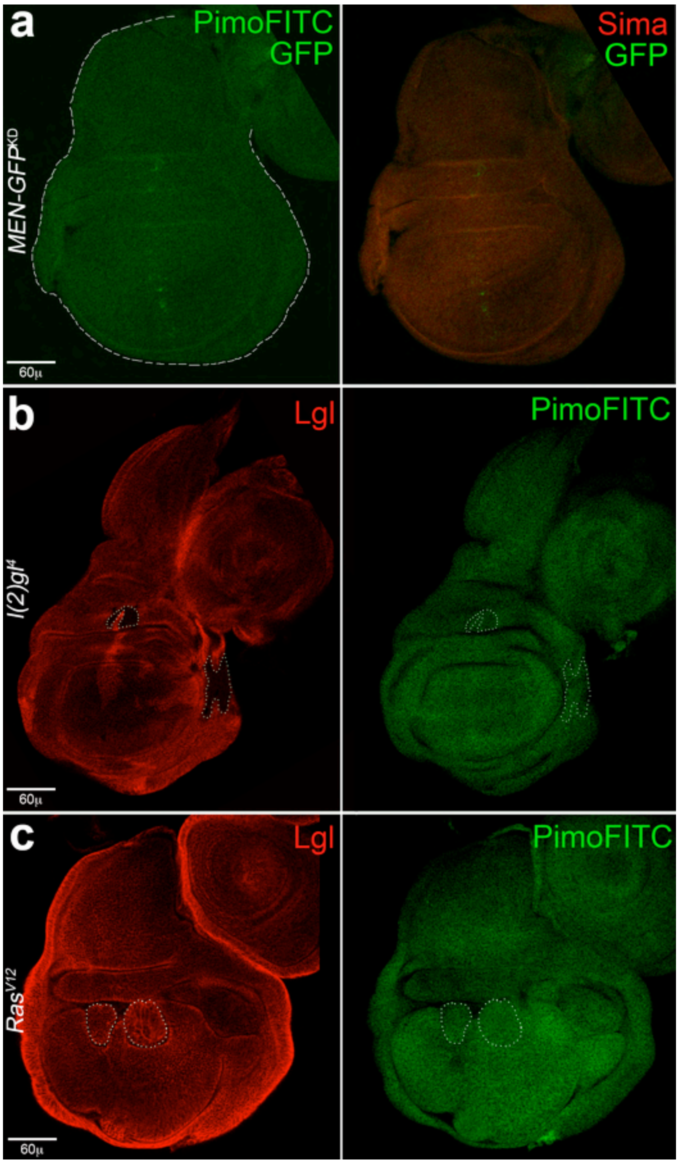

Supplementary Figure 3 | Pimonidazole control assays. (**a**) Imaginal wing discs from *w*; *Rpl27A*^{1}, *en-Gal4*, *UAS-GFP*/+; *UAS-GFP-RNAi*/+ individuals. The P compartment (to the left), identified thanks to a residual GFP fluorescence at the A/P border, does not suffer from hypoxia and Sima staining is uniform across the disc. (**b**) Imaginal wing discs bearing *l(2)gl*^{4} clones (Lgl^{-}, dotted in white) induced in *yw*, *hs-Flp, tub-Gal4*/+; *tub-Gal80, FRT40A*/*l(2)gl*^{4}, *FRT40A* individuals. (**c**) Imaginal wing discs bearing *Ras*^{V12} clones (dotted in white) induced in *yw*, *hs-Flp, tub-Gal4*/+; *tub-Gal80, FRT40A*/*FRT40A*; *UAS-Ras*^{V12}/+ individuals. Pimonidazole assays reveal that neither *l(2)gl*^{4} (**b**) nor *Ras*^{V12} (**c**) cells undergo hypoxic stress. Genotypes are indicated in each image.

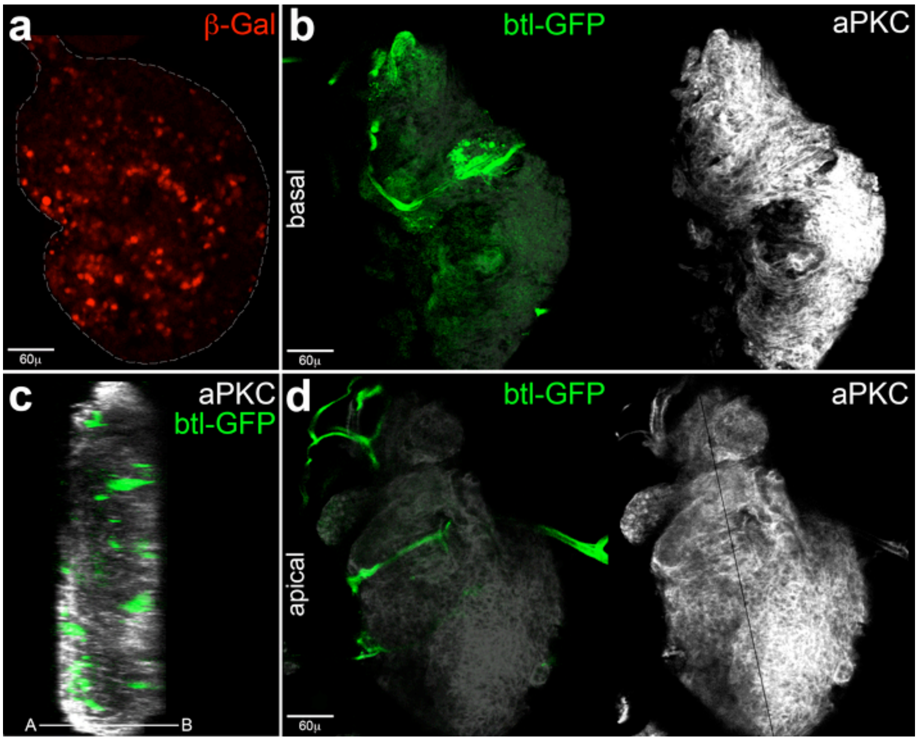

Supplementary Figure 4 | *l(2)gl^4* mutant discs show *bnl* promoter activation and ectopic tracheal branches. Imaginal wing discs from *w*; *l(2)gl^4*, *FRT40A*/*l(2)gl^4*, *FRT40A*; *bnl-LacZ*/*btl-Gal4*, *UAS-mCD8::GFP* individuals. (**a**) *bnl* promoter is active in a significant proportion of the mutant tissue. (**b-d**) Basal and apical sides of a mutant wing disc showing ectopic tracheal branches (GFP$^+$). In **c**, a section along the *z* axis reveals the presence of tracheal cells (GFP$^+$) in the disc depth. A: apical; B: basal. In **a**, disc contour is outlined in white.

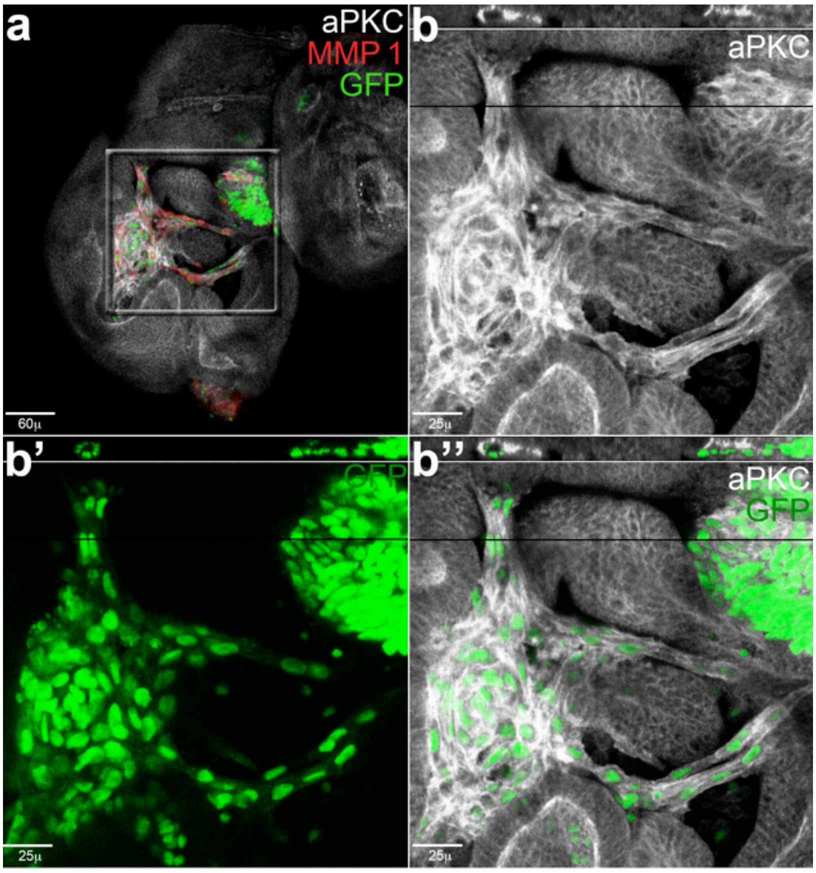

Supplementary Figure 5 | *l(2)gl*^{4}, *Ras*^{V12} cells form branched and tubule-shaped structures during cancer progression.

(**a**) An imaginal wing disc bearing *l(2)gl*^{4}*Ras*^{V12} clones induced in *yw*, *hs-Flp, tub-Gal4, UAS-GFP*/+; *tub-Gal80, FRT40A*/*l(2)gl*^{4}, *FRT40A*; *UAS-Ras*^{V12}/+ individuals. (**b-b''**) Magnifications of the region squared in (**a**) showing branched structures departing from the mutant clone. Migrating GFP^{+} cells are positive for the junctional marker aPKC and the *z* section shown in the upper part of the images reveals a tubule-shaped structure encircling a rudimental lumen.

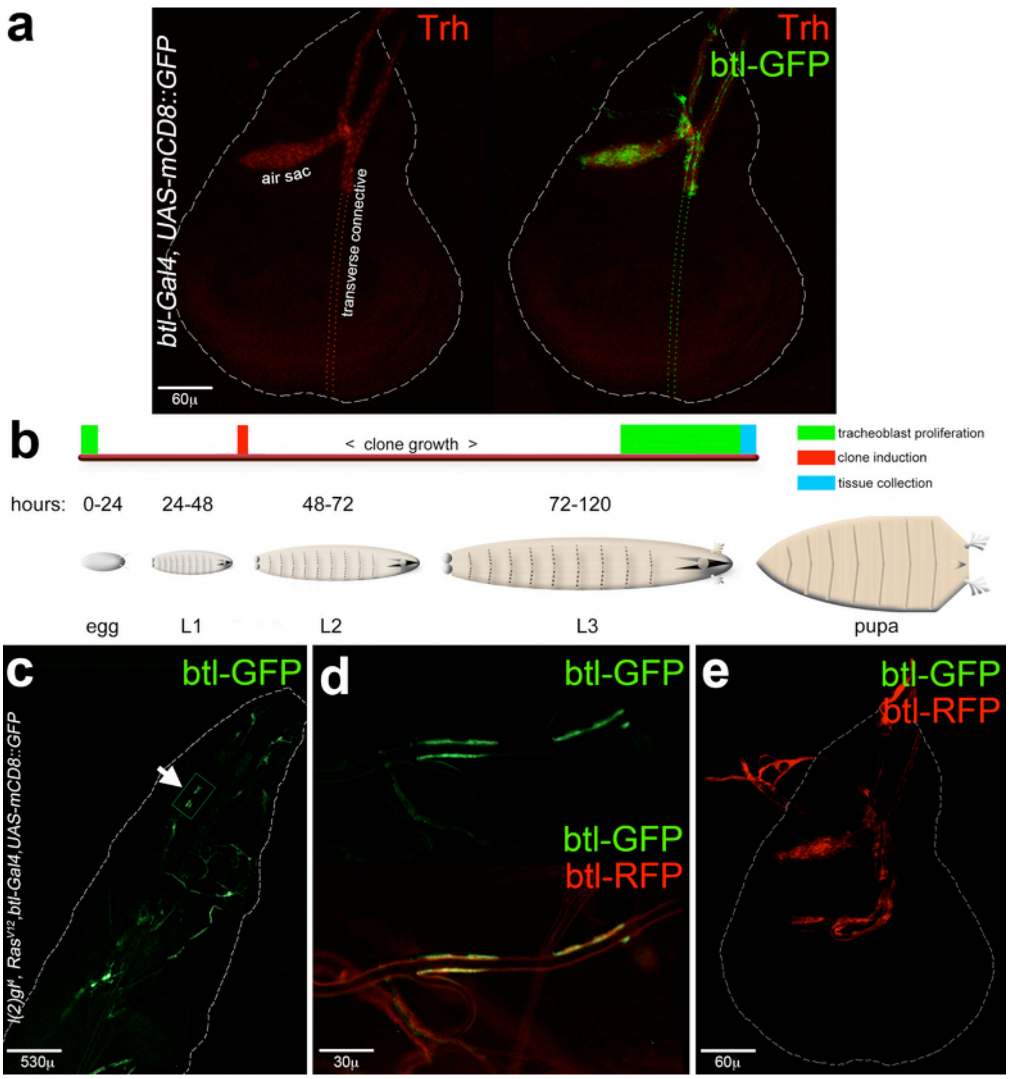

Supplementary Figure 6 | Epithelial-to-tracheal switch control experiment. (**a**) Tracheal fate determinants in imaginal wing discs from *w*; *btl-Gal4*, *UAS-mCD8::GFP* individuals: Trachealess co-localises with Btl/FGFR in the tracheal structures contacting the basal side of the disc; in particular, the air sac and the transverse connective are labelled, with the missing part of the latter dotted in red (left) and green (right). (**b**) Scheme describing the time frame for tracheoblast proliferation (green, 5-7 h AEL and from 85 h AEL onwards), clone induction (red, L1/L2 transition) and tissue collection (blue, 110-120 hours AEL) in wild-type individuals reared at 25°C. As can be seen, at clone induction tracheoblasts are quiescent, therefore not prone to mitotic recombination. (**c-e**) *l(2)gl*^{4}*Ras*^{V12} clones induced specifically in the tracheal tree of *hs-Flp*/+; *l(2)gl*^{4}, *FRT40A*/*tub-Gal80*, *FRT40A*; *btl-RFPmoe*, *btl-Gal4*, *UAS-mCD8::GFP*/*UAS-Ras*^{V12} individuals at 0-4 hours AEL (**c**,**d**) and at 48 ± 4 h AEL (**e**). In **c**, the anterior half of an L3 larva is shown bearing several tracheal clones (0-4 h AEL clone induction); in particular, the clone outlined in green in **c** (arrow) is magnified in **d**, with the tracheal branches marked by the *btl-RFPmoe* reporter. (**e**) A representative imaginal wing disc (48 ± 4 h AEL clone induction) is shown where no GFP^{+} cells are visible in the epithelium or in the tracheal structures. Genotypes are indicated in each image.

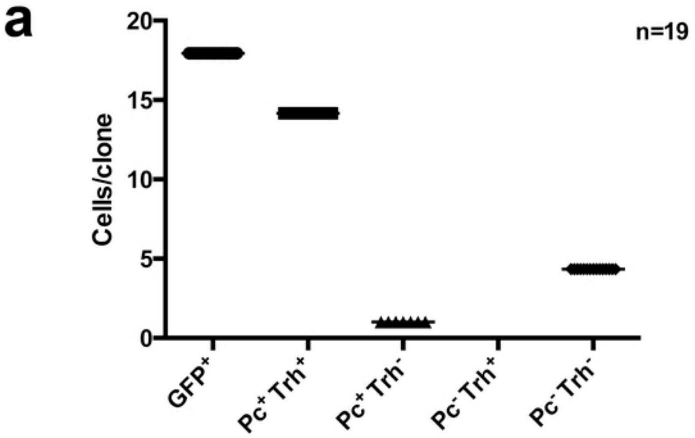

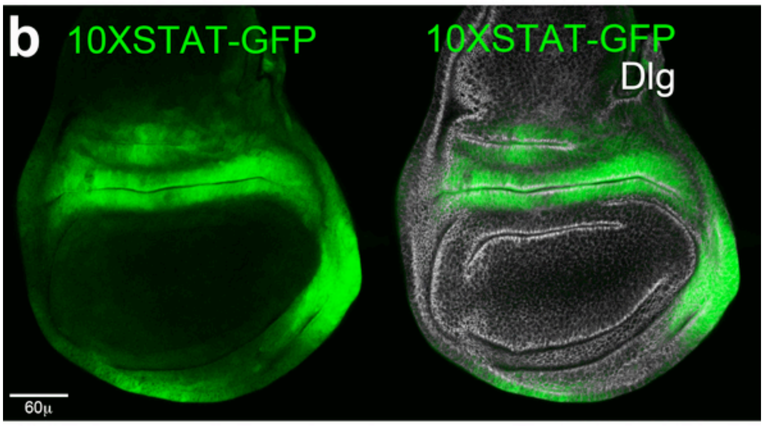

c

n=46

	Proximal	Distal
STAT92E$^+$ Trh$^+$	9	11
STAT92E$^-$ Trh$^-$	6	4
STAT92E$^+$ Trh$^-$	8	5
STAT92E$^-$ Trh$^+$	1	2

Supplementary Figure 7 | Pc/Trh and STAT92E/Trh co-localisation in *$l(2)gl^4Ras^{V12}$* clones. (**a**) Graph representing the average number of cells per clone scored for Pc$^+$ and Trh$^+$ expression. The analysis was carried out on mutant clones attached to or included in a tracheal vessel. In most cases, Pc and Trh are either both expressed or both not expressed. (**b**) Activation of the 10XSTAT-GFP construct in a *wild-type* background (future hinge); Dlg marks disc structure. (**c**) Table representing the number of mutant clones in the proximal and distal regions of the wing scored for STAT92E and Trh expression.

Supplementary Movie 1 | Basal-to-apical reconstruction of a trans-migration phenomenon. *MEN-lgl*^{KD} cells are shown while moving from the P compartment of the haltere disc towards the P compartment of the wing disc. The arrowheads indicate, in order of appearance, GFP-positive cells moving out of the haltere disc (arrow 1), migrating along the tracheal scaffold (arrow 2) and entering the P compartment of the wing disc (arrow 3). The polarity marker aPKC is shown in red.
