# Supplementary figures and images for: Multiple strategies of oxygen supply in Drosophila malignancies identify tracheogenesis as a novel cancer hallmark
Source: Sci Rep. 2015 Mar 12;5:9061. doi: 10.1038/srep09061 (PMC4357021; doi:10.1038/srep09061)

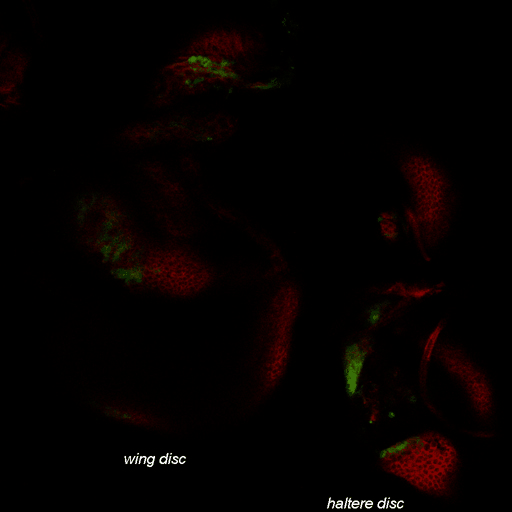

Supplement: Supplementary Information — Supplementary movie 1 [file srep09061-s2.gif]
